# Enhanced photoluminescence from CdS with SiO_2_ nanopillar arrays

**DOI:** 10.1038/srep11375

**Published:** 2015-06-16

**Authors:** Wei Li, Shaolei Wang, Sufeng He, Jing Wang, Yanyan Guo, Yufeng Guo

**Affiliations:** 1 College of Electronic Science and Engineering, Nanjing University of Posts and Telecommunications Nanjing, China 210003; 2 Key Laboratory of Radio Frequency and Micro-Nano Electronics of Jiangsu Province, Nanjing, China 210003; 3 Department of Electrical and Computer Engineering, University of California, San Diego, 9500 Gilman Drive, La Jolla, California 92093-0407, USA

## Abstract

In this paper, the enhanced photoluminescence from CdS thin film with SiO_2_ nanopillar array (NPA) was demonstrated. The CdS was prepared using chemical bath deposition in a solution bath containing CdSO_4_, SC(NH_2_)_2_, and NH_4_OH. The SiO_2_ NPA was fabricated by the nanosphere lithography (NSL) techniques. The nanopillar is about 50 nm in diameter, and the height is 150 nm. As a result, the sample with NPA shows an obvious improvement of photoluminescence (PL), compared with the one without NPA. In addition, we also observed that the PL intensity is increased ~5 times if the active layer is deposited on the nanopillar arrays and covered by a thin metal film of Al. It is noteworthy that the enhancement of photoluminescence could be attributed to the roughness of the surface, the 2D photonic band gap (PBG) effect and the surface plasmon resonance (SPR) effects.

The low-dimensional nanoscale semiconductor structures have been received much attention because of their anisotropic geometry results in unique physical properties, which offer great potential application^1^,^2^,^3^,^4^,^5^. Cadmium sulfide (CdS), an important II−VI semiconductor material, has a direct band gap of 2.45 eV at room temperature and is regarded as a possible optoelectronic material in the visible spectrum range^6^,^7^,^8^. Therefore, many researchers developed different techniques to control the shape and size of CdS nanostructures to improve the luminescence.

Nanosphere lithography is a well-know method to fabricate order nanopillar array^9^,^10^,^11^. This technique makes PS spheres to form large single or double layer, close packed arrays driven by capillary forces. Then, this array is applied as a mask. After RIE and subsequent lift-off, the large-scale ordered nanopillar arrays can be obtained. Meanwhile, the phenomenon of enhanced light emission has been reported by using photonic crystal (PC) patterns. For example, Nikhil Ganesh said that the fluorescence intensity can be enhanced because of PC structure^12^. Doo-Hyun Ko reported that the PC structure helps transcend the electrical performance constraints of a thin photoactive layer to improved light emission in organic solar cell^13^. Fuchyi Yang demonstrated that enhanced excitation effects were simultaneously possible at wide wavelength separation due to the 2D asymmetric PC design^14^. In this paper, photonics crystal patterns with photonic band gap are well fabricated by nanosphere lithography on the SiO_2_ substrate and the effects of such nanostructures on the optical properties have been studied.

In addition, the surface plasmon resonance effects of the metal can also improve the emission intensity. Jyh-Lih Wu blended gold nanoparticles into the anodic buffer layer and had a significant increase in fluorescence intensity^15^. Feng Wang achieved that the improvement on the luminescence efficiency by coupling between localized surface plasmons within silver nanostructures and excitons in a silicon-rich silicon nitride (SiN*x*) matrix^16^. Here, metal coved nanostructured has also been investigated. It is observed that about 5 times increase of photoluminescence emitted from this nanostructure.

## Sample preparation

**Figure 1:**
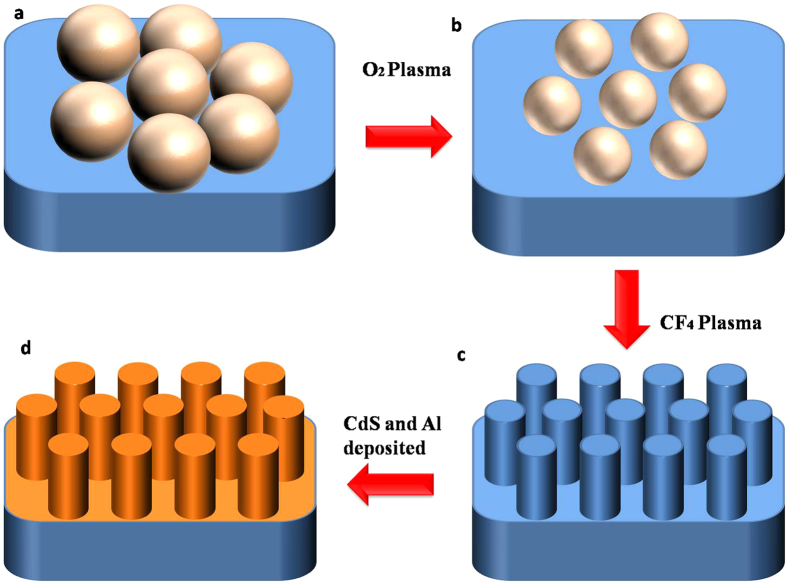
The schematics of the procedure for fabricating nanostructure.

In this experiment, we used nanosphere lithography method to fabricate nanopillar array^17^,^18^,^19^,^20^. Glass sheets coated with a SiO_2_ film of 300-nm thickness by PECVD are used as the substrates. Then, these substrates are coated with polystyrene (PS) sphere solution to form large area and hexagonally close packed (HCP) structures on the surface. By depending on the concentration, monolayer PS spheres can be obtained (Fig. 1a). And then, the prepared substrate with PS sphere was etched by reaction ion etching (RIE) with oxygen at these conditions: O_2_ flow, 20 sccm, RF power, 20 W, chamber pressure, 1 Torr. O_2_ plasma was applied to transform the closely packed PS nanosphere monolayer into arrays of separated nanospheres (Fig. 1b). In the next step, the left PS spheres with thinned diameter are served as masks. RIE with CF_4_ was employed to create SiO_2_ NPA at these conditions: CF_4_ flow, 20 sccm, O_2_ flow, 10 sccm, RF power, 40 W, chamber pressure, 1 Torr. By sonication in ethanol for a few minutes, the PS spheres were easily removed. And, the wafer with ordered SiO_2_ nanopillars was obtained, as shown in Fig. 1c. The sample was then mounted into a bath beaker. The CdS film was deposited on the substrate by using chemical bath deposition in a solution bath containing CdSO_4_ (0.2 M), SC(NH_2_)_2_ (0.5 M), and NH_4_OH (0.06 M). The deposition temperature was 60 °C and the PH value was 9. After 1 h later, the substrates were removed from the chemical bath, cleaned thoroughly in distilled water and dried in the air at room temperature. Finally, 30 nm thick Al was deposited on the top (Fig. 1d). It was prepared by electron-beam evaporation (EBV) and subsequently heat-treated at 350 °C for 10 min. The process of EBV was performed at a 5 × 10^−4^  Pa vacuum and the evaporation cur-rent was 25 mA. For comparison, the one without the nanopillar array was also fabricated.

## Results and Discussion

**Figure 2:**
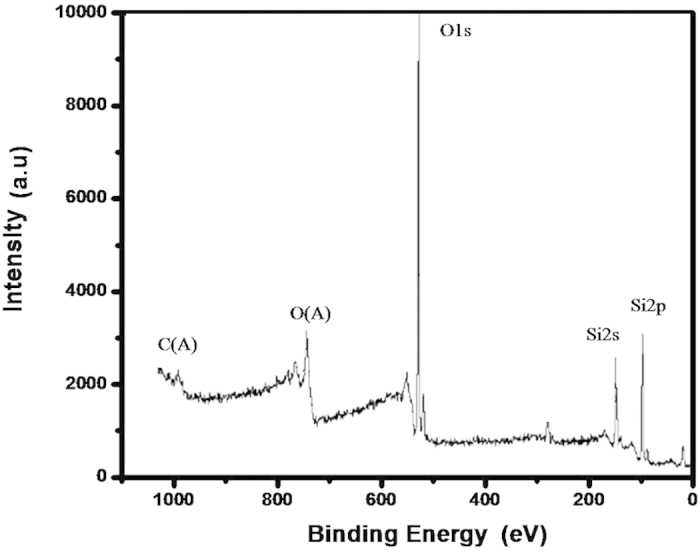
XPS spectra of SiO_2_.

**Figure 3:**
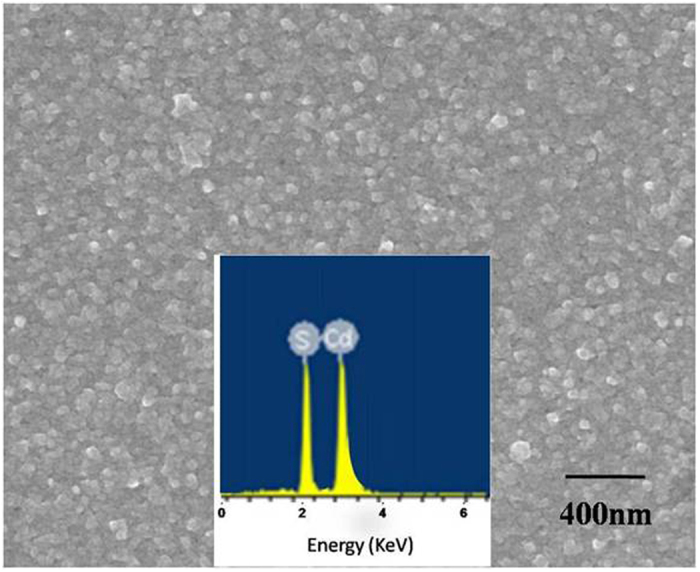
The SEM image of CdS film and the inset is the corresponding EDS recorded from the samples.

**Figure 4:**
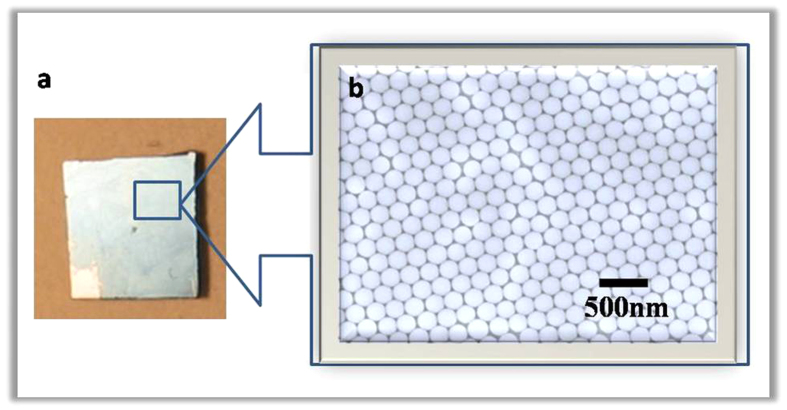
A photograph (**a**) and a SEM image of a monolayer PS spheres array (**b**).

**Figure 5:**
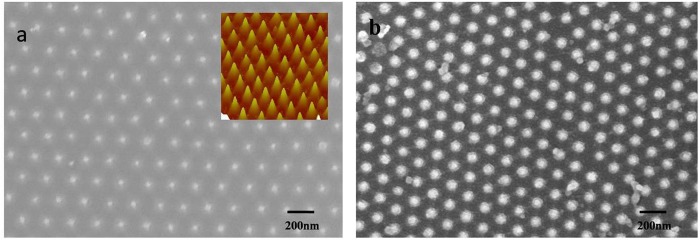
(**a**) SEM and AFM image of SiO_2_ nanopillar array. (**b**) SEM image of SiO_2_ nanopillar deposited by CdS.

We used SEM and AFM measurement to investigate the structure and the morphology of the SiO_2_ nanopillars. The SEM measurement was performed on LEO1530VP. And the AFM measurement was performed on Nanoscope III (Digital Instrument, USA). The elemental analyses of the samples were conducted with an energy dispersive X-ray spectrometer (EDS, BDX3200). Photoluminescence (PL) spectra were measured at room temperature by SPEX Flurolog-2 spectrofluorometer. The luminescent lifetime was measured by FS920. The result of XPS study of SiO_2_ film is shown in Fig. 2. As seen in Fig. 2, the peak of O1s is 530.2 eV, the peak of Si2p is 102.8 eV and the peak of Si2s is 154.1 eV. This is a typical electronic spectrum of elemental Oxygen and Silicon. The SEM image of CdS film is shown in Fig. 3. Here, the CdS film was growing on the substrate with nanoparticls size of 5 nm−100 nm.The inset in Fig. 3 shows the EDS of the corresponding sample, displaying the element of Cd and S in the samples. Figure 4a is a photograph of a monolayer PS spheres array with a 1 cm^2^ area. And a SEM micrograph is shown in Fig. 4b. As seen in this image, a uniform arrangement of the spheres with single layer is obtained in a large area. It is a HCP structure. Figure 5a shows a SEM image of the SiO_2_ NPA obtained by nanosphere lithography. The insert is the AFM image. As observed, the periodic pattern is accurately transferred onto the SiO_2_ substrate after RIE. The HCP lattice is quite uniform. The lattice constant is 220 nm, which corresponds to the diameter of the PS spheres. The mean diameter of the SiO_2_ pillars is about 50 nm measured on the top of the pillars. The height of the pillar is about 150 nm from AFM image. But, it is clearly observed that the side wall of the pillar is not vertical. The nanopillar is cone-type. The tilted wall of the nanopillars should be due to the fact that diameters of the PS spheres were reduced during dry etching. Figure 5b is a SEM image of SiO_2_ nanopillar covered by CdS film. It is found that these pillars are quite uniform and the lattice constant is still around 220 nm. Also, no pillars was changed their location.

**Figure 6:**
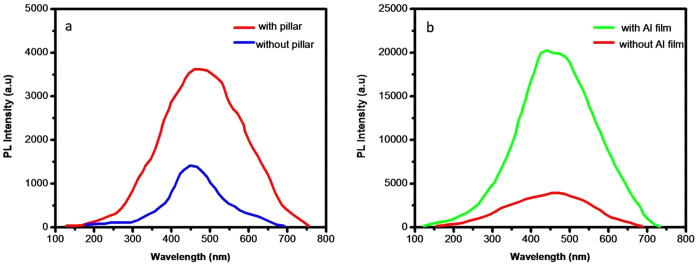
(**a**) PL spectra of the sample with and without SiO_2_ nanopillar arrays. (**b**) PL spectra of the sample with and without covering of Al film on the nanostructured.

**Figure 7:**
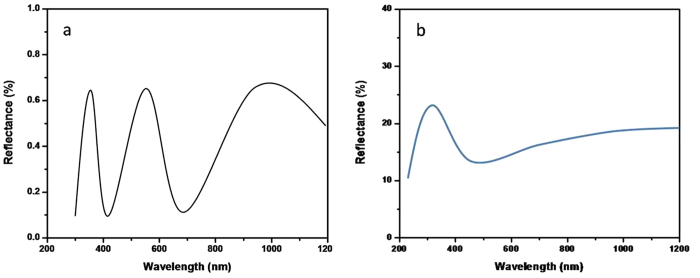
(**a**) Reflectance measurements of SiO_2_ nanopillar. (**b**) Reflectance measurements of SiO_2_ film.

He–Ge laser with 325 nm line was used as excitation source. The photoluminescence (PL) spectrum of the sample with and without SiO_2_ nanopillar is shown in Fig. 6a. The speak locates at around 450 nm. It is clearly observed that the PL intensity of the sample with the SiO_2_ nanopillar arrays is increased about 2.3 times comparing with that without nanopillar nanostructures. The increase of PL intensity may be explained by two mechanisms. One is the roughness of the surface formed by the nanostructures of the sample. It will change the reflection of the sample and make more light extraction. As discussed above, the side walls of the nanopillars are not vertical as seen in Fig. 5a. It is found that the formed SiO_2_ nano-structures have the cone-shape which causes the gradually increase of fractional area occupied by SiO_2_ from top to bottom. And the gradually changing reflective index from the surface can effectively eliminate the reflected light across a wide spectrum due to the reflective index matching. The gradually changing of SiO_2_ filling factor can result in the formation of gradual refraction index from front surface to bottom as in our case. Therefore, the reflection of the front surface can be significantly reduced. Figure 7(a) is the reflection spectrum of the SiO_2_ NPA. It is shown that the reflectivity is reduced to lower than 1%. Figure 7(b) is the reflection of the SiO_2_ film. The reflectivity from the front surface of SiO_2_ film is quite high in the whole measurement range and comes up to higher than 15%. It is obviously that the reflection is suppressed for all the nano-patterned samples.

**Figure 8:**
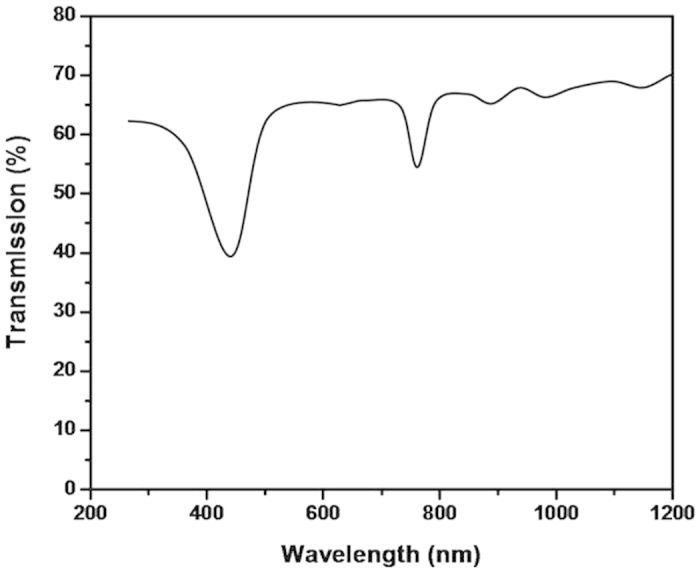
The transmittance spectra of SiO_2_ NPA.

In the other hand, the 2D PBG effect may also play an important role for extracting light from the periodic ordered structures. Figure 8 shows the transmittance spectra of the SiO_2_ NPA. It is found that there is a band gap of 452 nm for the SiO_2_ nanopillar. It is very similar to the PL peak of the sample. It is reported there are two possible ways to enhance light extraction by 2D PBG effect^21^,^22^: (1) because multiple scattering of photons by lattice of periodically varying refractive indices in the PCs acts to form PBGs in which lateral propagation of the Bloch guided modes is prohibited, light generated in the band gap region can couple only to radiation modes and is radiated outward. (2) the refractive index periodicity creates a cutoff frequency for guided modes. Guided modes are folded by the PCs at the Brillouin zone boundaries, allowing phase matching to the radiation modes that lie above this cut-off frequency. The guided modes that phase match to the radiation modes become leaky resonances of the PCs which Bragg scatter the light emitted from of the active region.

**Figure 9:**
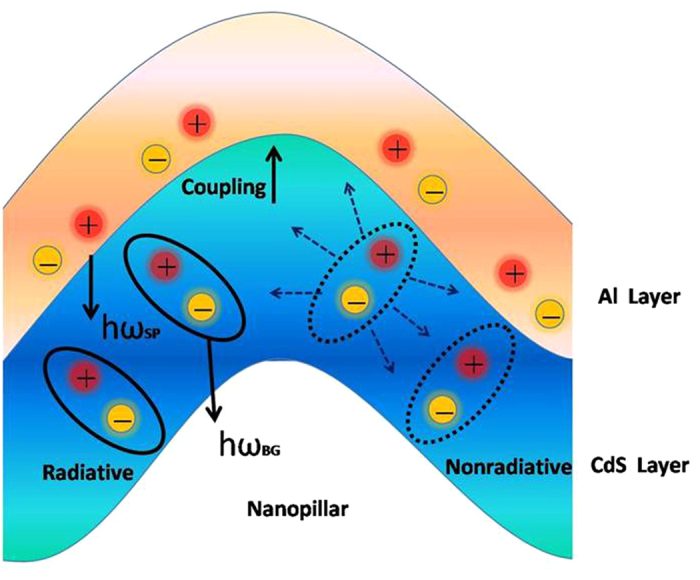
Schematic diagram of the electron–hole recombination and SPR coupling mechanism.

Figure 6(b) shows the PL results of the samples (with nanopillar) with and without Al film depositing on the nanostructure. It is clearly observed that PL intensity of the sample with metal covered is significantly increased. It is about 5 times than that without metal covered. One possible mechanism is the effect of SPR^23^,^24^. In general, the surface plasmon resonance improves the luminescence properties due to following two reasons: firstly, the coupling between SPR and active centers increase the radiative transition rates; and secondly, plasmonic nanostructures can concentrate the incoming light into strong localized electric fields. There is a possible explanation of the CdS–SP coupling and the light extraction shown in Fig. 9. First, electron–hole pairs are generated in the CdS layer by photo pumping. For the samples without metal coated, these carriers are terminated by the radiative (k_rad_) or nonradiative (k_non_) recombination rates, and the internal quantum efficiency (IQE,η) is determined by the ratio of these two rates:


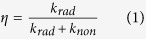


When a metal layer is grown on the active layer and the bandgap energy (h*ω*_BG_) of CdS layer is close to the electron oscillation energy (h*ω*_SP_) of SP at the metal/semiconductor surface, the CdS energy can transfer to the SP. PL decay rates are enhanced through the CdS–SP coupling rate (*k*_SPC_). Under the existence of the SP coupling, the enhanced IQE of emission(η^*^) can be described as follows:


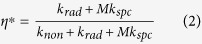


**Figure 10:**
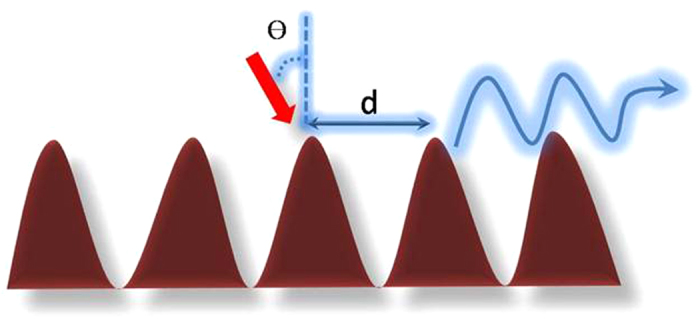
Schematic diagram of plasmonic nanostructures mechanism.

Where M is the probability of photon extraction from the SPs energy and should depend on the roughness and nanostructure of the metal surface. When the SP coupling rate *k*_SPC_ is much faster than *k*_rad_ and *k*_non_, the IQE should be dramatically increased. And there is another interpretation of plasmonic nanostructures shown in Fig. 10. It is that metal-covered diffraction grating couplers provide a method to excite plasmons, as multiple diffracted orders each excite plasmons based on the following matching condition^25^:


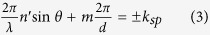


**Figure 11:**
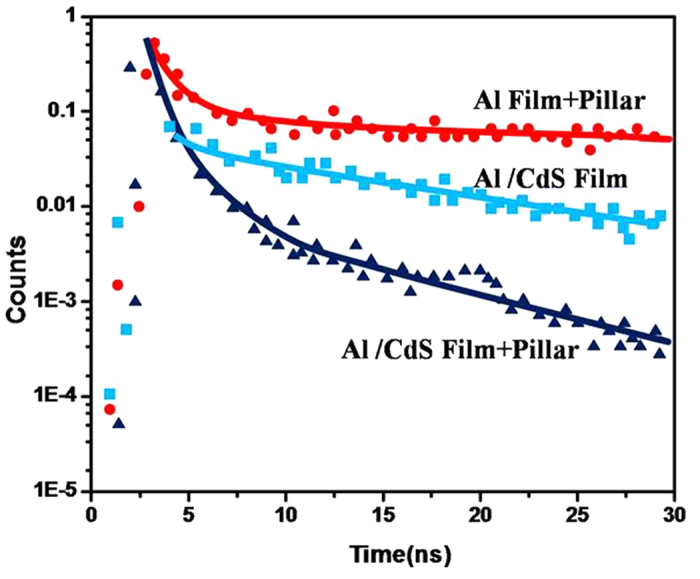
The fluorescence decay curves of (**a**) Al filme with SiO_2_ NPA, (**b**) Al(covered)/CdS, (**c**) Al (covered)/ CdS with SiO_2_ NPA.

Here n’ is the refractive index of the dielectric, m is the diffracted order (0, ±1, ±2…), and d is the grating pitch. To distinguish these two mechanisms, the analysis of luminescent lifetime on various samples was measured. Figure 11 shows the fluorescence decay curves, with peaks normalized to unity, for three samples: (a) Al filme with SiO_2_ NPA, (b) Al(covered)/CdS and (c) Al (covered)/ CdS with SiO_2_ NPA. It is interesting to find that the intensity decay of the sample c is faster than that of others. The intensity-decay data were fit to the multi-exponential model where the intensity decay is given by^26^:


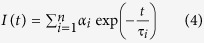


where α_i_ is amplitude factors associated with each decay time τ_i_. It is enhanced by 2 fold than the sample b and 3 fold than the sample a. In our experiments, the emission efficiency can be further increased by decreasing the metal film thickness and replacing Al to less lossy metal such as Ag and etc. These will be reported in the next paper.

## Conclusion

In summary, nanosphere lithography is successfully used to create the nanopatterns on SiO_2_ substrates. The enhanced photoluminescence from the CdS thin film with SiO_2_ NPA was demonstrated. In addition, 5 times increase of photoluminescence emitted from this nanostructure with Al covering has been observed. The main mechanism of enhancement is due to the roughness of the surface, the 2D photonic band gap PBG effect and the surface plasmon resonance effects. Further improvements can be obtained by optimizing the design of the nanostructure geometry and using less lossy metal.

## Additional Information

**How to cite this article**: Li, W. *et al.* Enhanced photoluminescence from CdS with SiO_2_ nanopillar arrays. *Sci. Rep.* **5**, 11375; doi: 10.1038/srep11375 (2015).
